# The Mediator Complex: From Transcriptional Regulation to Disease Pathogenesis

**DOI:** 10.3390/ijms27052221

**Published:** 2026-02-26

**Authors:** Sailakshmi Iyer, Takashi Ito, Takeya Nakagawa, Naoko Hattori

**Affiliations:** 1 Department of Biochemistry, Graduate School of Biomedical Sciences, Nagasaki University, Nagasaki 852-8523, Japan; 2 Department of Dermatology, Nagasaki Graduate School of Biomedical Sciences, Nagasaki University, Nagasaki 852-8523, Japan

**Keywords:** mediator complex, cancer, MED19, PPI, chromatin

## Abstract

The Mediator complex is a central regulator of eukaryotic transcription, functioning as a dynamic molecular interface between gene-specific transcription factors and RNA polymerase II (Pol II). Although its overall architecture and general role in transcription have been extensively reviewed, accumulating genetic, genomic, and clinical evidence indicates that individual Mediator subunits make distinct and non-redundant contributions to human physiology and disease. In this review, we move beyond a generic description of Mediator function and present a subunit-resolved synthesis of Mediator biology with an emphasis on disease pathogenesis. A key feature of this review is a comprehensive table integrating disease associations and molecular functions of individual human Mediator subunits, enabling rapid assessment of functional specialization across the complex. We further discuss chromatin-based mechanisms of Mediator action, including cooperation with cohesin and architectural factors to regulate enhancer-promoter communication and higher-order genome organization. By organizing recent structural, mechanistic, and pathological findings into a unified framework, this review highlights how disruption of specific Mediator subunits contributes to cancer, developmental disorders, and metabolic disease, and outlines emerging opportunities for therapeutic intervention.

## 1. The Mediator Complex in Transcriptional Regulation

The Mediator complex lies at the very heart of eukaryotic gene regulation, coordinating communication between DNA-bound transcription factors (TFs) and RNA polymerase II (Pol II). This multi-subunit complex was originally identified through pioneering studies investigating how TFs regulate Pol II–mediated transcription in yeast [1,2,3,4]. The Mediator complex is evolutionarily conserved from yeast to humans, yet its subunit composition and primary amino-acid sequences show substantial divergence across species. In Saccharomyces cerevisiae, Mediator consists of approximately 25 subunits and has an estimated molecular mass of ~0.8 MDa, whereas the human Mediator complex comprises roughly 30 subunits and reaches ~1.4 MDa in size [5,6].

Over the past decade, advances in structural, biophysical, and functional analyses have fundamentally reshaped our understanding of Mediator. Rather than functioning as a static coactivator, Mediator is now recognized as a dynamic, centralized, and signal-responsive regulatory hub [7]. Groundbreaking cryogenic electron microscopy (cryo-EM) studies—including near-atomic-resolution models of the Mediator–Pol II preinitiation complex (PIC)—have revealed remarkable structural flexibility and a modular architecture in which the kinase, head, middle, and tail modules undergo coordinated rearrangements to promote transcription initiation [5,8].

Together, these findings establish the Mediator complex as a dynamic integrator of transcriptional regulation whose roles extend well beyond traditional models. In this review, we summarize recent advances in defining the modular organization and functional versatility of Mediator, with a particular focus on its roles in development and disease. We also discuss emerging therapeutic directions that have gained attention over the past decade, including inhibition of the Mediator kinase module, disruption of Mediator-centered protein–protein interactions (PPIs), and growing evidence that mediator complex subunit 19 (MED19) represents a potential transcriptional vulnerability in cancer.

This review was conducted as a scoping analysis aimed at capturing the breadth of current evidence on Mediator complex structure, function, disease relevance, and therapeutic potential, with a primary focus on advances reported between 2015 and 2025.

## 2. Structural Organization and Modular Architecture

Recent advances in cryo-electron microscopy (cryo-EM) and X-ray crystallography have transformed our understanding of the Mediator complex, revealing its evolution from a putative scaffold into a highly intricate and dynamic modular assembly. Mediator is organized into four major modules—Head, Middle, Tail, and Kinase—each contributing distinct structural and regulatory functions. A central architectural feature of the complex is mediator complex subunit 14 (MED14), which spans nearly the entire assembly. MED14 acts as a structural backbone, tightly linking the head and middle modules while anchoring the tail module at the base of Mediator. Although the precise modular assignment of MED14 remains debated, we consider MED14 as a subunit of the tail module in accordance with the reference [6].

The head and middle modules, together with MED14, form the so-called core Mediator (cMed). Within this core, MED14 is indispensable for robust interaction with RNA Pol II. While the head and middle modules alone can assemble into a stable complex, association with MED14 is required to reconstitute a fully functional Mediator capable of productive transcriptional engagement. In contrast, the kinase module—commonly referred to as the CDK8 kinase module (CKM)—associates with Mediator in a reversible manner, providing an additional layer of regulatory flexibility [9,10,11].

High-resolution structural insights have been instrumental in defining functional interfaces within Mediator. The 3.4 Å crystal structure of *Schizosaccharomyces pombe* cMed, encompassing the head and middle modules, revealed that multiple disease-associated mutations cluster at the interface between mediator complex subunit 6 (MED6) and mediator complex subunit 17 (MED7), which connect to the middle module. This clustering highlights critical structural hotspots essential for Mediator integrity and activity [8]. More recently, near-atomic-resolution cryo-EM structures have resolved nearly the entire tail module, as well as MED1-containing regions of the middle module, providing direct visualization of how transcriptional activator binding and Pol II engagement induce long-range conformational changes that propagate from the head to the tail modules [12].

**Figure 1:**
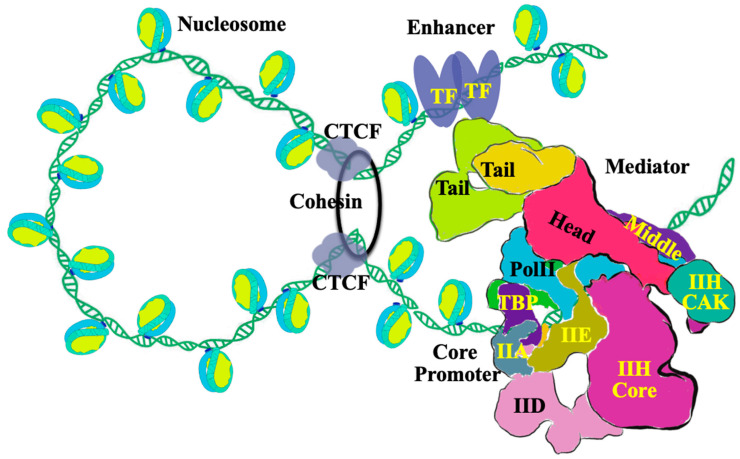
Mediator-centered transcriptional regulatory network at promoters and enhancers. Schematic representation of Mediator-dependent transcriptional regulation integrating transcription factors (TFs), chromatin architecture, and the basal transcription machinery. Sequence-specific TFs bind enhancer regions and interact with the Tail module of the Mediator complex (shown in light green and yellow), facilitating signal-dependent recruitment and activation. Mediator cooperates with cohesin and CTCF to promote enhancer-promoter communication through chromatin looping and higher-order genome organization. At core promoter regions, Mediator assembles with the preinitiation complex (PIC), which includes general transcription factors and RNA polymerase II (Pol II), to form a Mediator-PIC (PIC–MED) assembly that supports transcription initiation. The α-helix bundle of the Head module (shown in red) and the Knob domain of the Middle module (shown in purple) form a Head–Middle interface that stabilizes the C-terminal domain (CTD) of RNA polymerase II. The structural organization of the human Mediator and Mediator-bound PIC is based on cryo-EM structures (Chen et al., 2021) [13], illustrating the spatial coordination between TF-bound Mediator, the core promoter, and the basal transcription machinery.

Mediator is now recognized to adopt at least two major conformational states: an extended and a bent configuration. Notably, the preinitiation complex (PIC) preferentially engages Mediator in the bent conformation, indicating that PIC architecture itself induces Mediator structural rearrangement [13]. Complementary high-resolution cryo-EM analyses of yeast Mediator–PIC assemblies further demonstrate that PIC binding drives precise reorganization of the Middle module, particularly within the hook, knob, and beam regions [14]. These structural studies establish Mediator as a conformationally dynamic complex whose architecture is actively reshaped during transcription initiation rather than functioning as a static scaffold [15] as outlined in Figure 1.

## 3. The Many Roles of the Mediator Complex

The Mediator complex plays numerous essential roles in eukaryotic transcriptional regulation, acting as a central integrator of regulatory signals throughout the transcription cycle. At the onset of transcription, Mediator functions as a molecular bridge that connects DNA-bound transcription factors with RNA Pol II, thereby facilitating assembly of the PIC [16]. Beyond this bridging role, Mediator actively stimulates transcription by promoting phosphorylation of the C-terminal domain of Pol II, leading to increased basal transcriptional output [4].

Mediator also plays critical roles during transcriptional elongation and termination. In particular, mediator complex subunit 26 (MED26) serves as a docking platform for elongation-associated factors, enabling efficient transition of Pol II into productive elongation [17]. In parallel, MED26 contributes to transcription termination by engaging the little elongation complex (LEC), thereby ensuring efficient and coordinated Pol II release at gene ends [18].

In addition to its canonical functions, Mediator has been implicated in higher-order mechanisms of transcriptional regulation. The formation of Mediator-containing biomolecular condensates at clusters of enhancers has been proposed as a mechanism to concentrate transcriptional machinery and enhance gene activation [19,20,21]. Moreover, the reversible association of the kinase module with Mediator allows cellular signaling pathways—including metabolic and developmental cues—to be integrated into transcriptional outputs by modulating enhancer activity, Pol II elongation, and chromatin organization [22].

Mediator is preferentially recruited to enhancer regions, where it serves as a central integrator of transcriptional regulation [23]. At enhancers, sequence-specific transcription factors (TFs) engage the Tail module of Mediator, promoting signal-dependent recruitment and activation. Mediator cooperates with cohesin to facilitate enhancer–promoter looping, a higher-order chromatin configuration essential for robust gene activation [24]. Consistent with this model, acute Mediator depletion reduces enhancer–promoter contacts, diminishes cohesin occupancy at enhancers, and decreases transcriptional output [25]. In addition, Mediator interfaces with CTCF-bound architectural elements to coordinate cohesin loading and loop extrusion, thereby contributing to chromatin domain organization [25,26]. At core promoter regions, Mediator assembles with general transcription factors and RNA polymerase II (Pol II) to form the Mediator–PIC (PIC–MED) complex, whose structural organization has been elucidated by cryo-EM [13]. Together, these findings position Mediator as a molecular platform that links higher-order chromatin architecture at enhancers with transcription initiation at promoters (Figure 1).

These mechanisms are particularly prominent in pluripotent embryonic stem cells (ESCs), where master transcription factors bind enhancer elements and recruit Mediator to activate cell identity gene programs. This process underscores the critical role of super-enhancers in maintaining mammalian cell fate and transcriptional robustness [27].

## 4. Mediator Dysregulation in Disease: From Modular Molecular Function to Vulnerability

**Figure 2:**
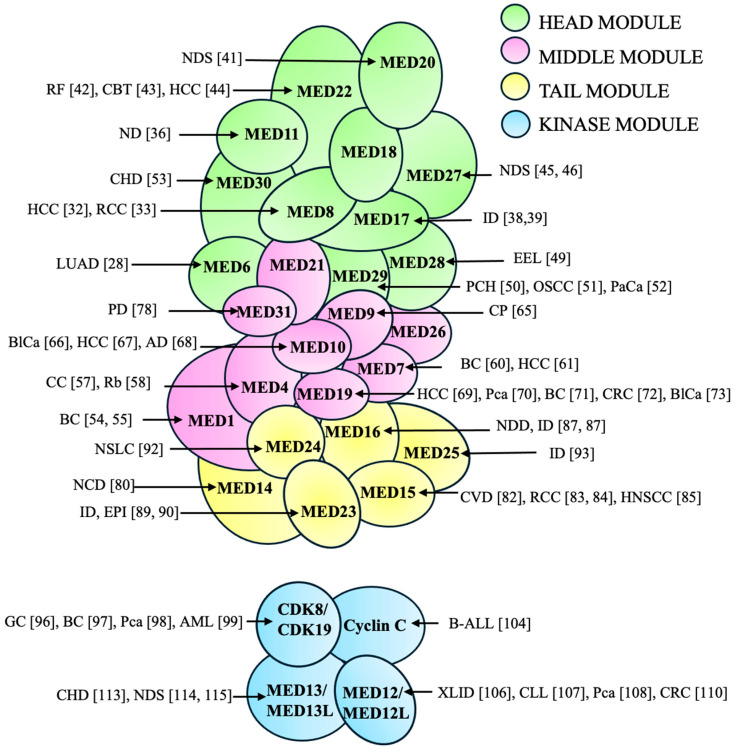
Disease-associated landscape of the human Mediator complex. Schematic representation of the Mediator complex showing the spatial organization of its four modules Head (green), Middle (pink), Tail (yellow), and Kinase (blue), and the association of individual subunits with human diseases. Representative disease associations are indicated in the figure. The numbers shown in brackets correspond to the reference list. Abbreviations: AD, Alzheimer’s disease; AML, acute myeloid leukemia; B-ALL, B-cell acute lymphoblastic leukemia; BC, breast cancer; BlCa, bladder cancer; CBT, carotid body tumor; CC, cervical cancer; CHD, congenital heart defects; CP, cardiac pathology; CRC, colorectal cancer; CVD, cardiovascular disease; EEL, early embryonic lethality; EPI, epilepsy; GC, gastric cancer; HCC, hepatocellular carcinoma; HNSCC, head and neck squamous cell carcinoma; ID, intellectual disability; LUAD, lung adenocarcinoma; NCD, neural crest development defect; ND, neurodegenerative disorders; NDD, neurodevelopmental delay; NDS, neurodevelopmental syndromes; NSCLC, non–small cell lung cancer; OSCC, oral squamous cell carcinoma; PaCa, pancreatic cancer; PCa, prostate cancer; PCH, pontocerebellar hypoplasia; PD, Parkinson’s disease; RCC, renal cell carcinoma; RF, renal failure; Rb, retinoblastoma; XLID, X-linked intellectual disability syndromes.

Disease phenotypes are rarely caused by complete loss of the Mediator complex. Instead, pathological states typically arise from selective dysfunction or hijacking of specific Mediator subunits distributed across its four modules. Accumulating genetic, genomic, and functional studies indicate that individual Mediator subunits constitute discrete points of vulnerability, giving rise to diverse disease manifestations. Below, we summarize disease associations linked to Mediator subunits, organized by module as illustrated in Figure 2 and summarized in Table 1.

### 4.1. Head Module

Over the past decade, the Mediator head module—which comprises mediator complex subunits MED6, MED8, MED11, MED17, MED18, MED20, and MED22, as well as proximally tethered subunits MED27, MED28, MED29, and MED30—has emerged as a major vulnerability center in a wide range of human diseases.

#### 4.1.1. Mediator Complex Subunit 6 (MED6)

MED6 has emerged as a potential predictive biomarker in lung adenocarcinoma (LUAD). A recent multi-omics analysis demonstrated that MED6 promotes tumor cell proliferation and is associated with poor prognosis, and identified drugs such as paclitaxel, afatinib, and brivanib as candidate therapeutic agents for MED6-high tumors [28]. Structurally, MED6 is a head-module subunit of the Mediator complex that contributes to RNA polymerase II C-terminal domain engagement [29]. Consistent with this positioning, foundational genetic and biochemical studies in Saccharomyces cerevisiae established MED6 as a selective regulator of activated, but not basal, Pol II transcription, functioning in pre-initiation complex formation and activator signal relay through functional interaction with SRB4 [30,31]. These conserved molecular functions provide a mechanistic basis for how MED6 dysregulation may preferentially amplify oncogenic transcriptional programs in LUAD.

#### 4.1.2. Mediator Complex Subunit 8 (MED8)

MED8 is frequently overexpressed in hepatocellular carcinoma (HCC), where elevated expression correlates with adverse clinical outcomes, and its depletion suppresses tumor cell proliferation, migration, and colony formation; accordingly, a MED8-centered immunomodulatory prediction model improves survival stratification of HCC patients [32], and MED8 has also been implicated in renal cell carcinoma (RCC) [33]. Mechanistically, MED8 is a conserved Mediator head-module subunit that forms a MED8/18/20 subcomplex engaging TBP and RNA polymerase II to promote pre-initiation complex formation and integrate activating and repressing transcriptional inputs, providing a molecular basis for its vulnerability in oncogenic transcriptional regulation [34,35].

#### 4.1.3. Mediator Complex Subunit 11 (MED11)

Homozygous mutations in MED11 cause severe neurodevelopmental impairment characterized by myoclonic seizures and premature death, and zebrafish models recapitulate these phenotypes, providing strong in vivo evidence for MED11’s essential developmental role [36]. Consistent with this requirement, yeast genetic and biochemical studies place MED11 (Srb4) in a Mediator subcomplex with MED6 that is critical for activated RNA polymerase II transcription and relay of activator signals to the basal transcription machinery [37].

#### 4.1.4. Mediator Complex Subunit 17 (MED17)

Biallelic variants in MED17 are associated with progressive microcephaly, intellectual disability (ID), and seizures. Patient-derived fibroblasts harboring MED17 mutations exhibit pronounced activation of the unfolded protein response, indicating proteostasis stress as a key pathogenic mechanism [38,39]. In this functional context, MED17 is a core component of the Mediator head module that supports RNA polymerase II-dependent transcription through direct interactions with general transcription factors and TFIIH, suggesting that loss of MED17 may broadly compromise transcriptional homeostasis in cells with high proteostatic demand [40].

#### 4.1.5. Mediator Complex Subunit 20 (MED20)

Mutations in MED20 cause infantile basal ganglia degeneration, a neurodegenerative disorder marked by cerebellar atrophy and basal ganglia abnormalities detectable by MRI [41]. Functionally, MED20 is a component of a conserved MED8/MED18/MED20 subcomplex within the Mediator head module that contributes to transcription initiation by forming a structural platform involved in TBP engagement and pre-initiation complex assembly, providing a plausible cellular context for the neurological vulnerability observed upon MED20 dysfunction [34].

#### 4.1.6. Mediator Complex Subunit 22 (MED22)

The strongest functional evidence for MED22 in human disease comes from podocyte-specific Med22 knockout mice, which develop progressive glomerular disease and succumb to renal failure, highlighting a critical role for Med22 in kidney physiology [42]. In cancer, proteomic analyses of carotid body tumors identified MED22 as a differentially expressed protein [43]. In addition, MED22—together with MED10 and meiosis-arrested-at-leptotene-1 (MEL1)—was found to be upregulated in HCC, forming a three-gene prognostic signature predictive of patient outcome [44]. Functionally, MED22 forms an essential heterodimer with MED11 within the Mediator head module, creating a conserved four-helix bundle that stabilizes the transcription pre-initiation complex through interaction with MED17 and is required for efficient Pol II transcription, providing a structural framework that may underlie its tissue-specific and disease-associated roles [37].

#### 4.1.7. Mediator Complex Subunit 27 (MED27)

Loss-of-function variants in MED27 cause an autosomal recessive neurodevelopmental syndrome characterized by global developmental delay, dystonia, cerebellar hypoplasia, spasticity, and cataracts. Zebrafish loss-of-function models failed to rescue developmental defects and revealed disruption of downstream transcription factor networks, including *foxo3a* and *fosab*, which have well-established roles in neurodevelopment, as demonstrated in zebrafish, mouse, and human studies [45,46]. Functionally, MED27 is an Upper Tail subunit of mammalian Mediator that forms extensive contacts with the core complex, as revealed by cryo-EM analyses, positioning it to modulate Mediator conformation and its interaction with RNA polymerase II [47]. Consistent with this architectural role, cardiomyocyte-specific ablation of Med27 in mice causes embryonic lethality or adult-onset heart failure and is accompanied by global destabilization of Mediator subunits, indicating that MED27 is essential for maintaining Mediator integrity in cardiac tissue independently of MED30 [48].

#### 4.1.8. Mediator Complex Subunit 28 (MED28)

Mouse knockout studies demonstrate that MED28 is essential for peri-implantation development and maintenance of pluripotency. Loss of Med28 results in early embryonic lethality accompanied by reduced expression of pluripotency regulators POU domain, class 5, transcription factor 1 (OCT4) and Nanog homeobox (NANOG) [49]. Functionally, MED28 is a metazoan-specific Tail-module subunit that participates in extensive contacts between the Tail and core Mediator, as revealed by mammalian cryo-EM structures, positioning it to influence Mediator conformation and transcriptional programs critical for early embryonic cell states [47].

#### 4.1.9. Mediator Complex Subunit 29 (MED29)

A 2025 study identified biallelic MED29 variants as the cause of pontocerebellar hypoplasia with cataracts, establishing MED29 as a novel risk gene for this disorder [50]. In oncology, MED29 expression is elevated in oral squamous cell carcinoma (OSCC), where it promotes epithelial–mesenchymal transition (EMT); MED29 knockdown suppresses epithelial–mesenchymal transition (EMT) and cell migration [51]. MED29 has also been shown to play context-dependent oncogenic and tumor-suppressive roles in pancreatic cancer (PaCa) [52]. In structural terms, MED29 is a metazoan-enriched Tail-module subunit embedded within the mammalian Mediator Tail that makes extensive contacts with the core complex, suggesting a role in shaping Mediator conformation and signal-dependent transcriptional outputs in a tissue- and context-specific manner [47].

#### 4.1.10. Mediator Complex Subunit 30 (MED30)

MED30 deletions have been linked to congenital disorders, including Langer–Giedion syndrome and Cornelia de Lange syndrome, underscoring its importance in human development [53]. Structurally, MED30 is a metazoan-specific proximal Tail subunit that anchors the Tail to the Head and core Mediator, as revealed by mammalian cryo-EM analyses, positioning it to influence overall Mediator architecture and signal integration [47]. Functionally, cardiomyocyte-specific deletion of Med30 in mice causes embryonic or adult-onset heart failure accompanied by destabilization of core Mediator subunits, demonstrating an essential role for MED30 in maintaining Mediator integrity and cardiac transcriptional networks in vivo [53].

Together, these studies demonstrate that although the Mediator head module functions as a cohesive structural entity, its individual subunits display distinct and disease-specific vulnerabilities. These vulnerabilities manifest as cancer, neurodevelopmental disorders, renal disease, or congenital syndromes. This emerging modular disease map argues strongly for subunit-specific therapeutic strategies, rather than generalized approaches to Mediator dysregulation.

### 4.2. Middle Module

The Mediator middle module comprises mediator complex subunits MED1, MED4, MED7, MED9, MED10, MED19, MED21, MED26, and MED31. This module functions as both a structural scaffold and a regulatory hub within the Mediator complex. Although these subunits are integrated into a single architectural unit, accumulating evidence indicates that they play distinct and sometimes divergent roles in human disease.

#### 4.2.1. Mediator Complex Subunit 1 (MED1)

MED1 is frequently amplified or overexpressed in cancer, most notably within the human epidermal growth factor receptor 2 (HER2) amplicon in breast cancer (BCa). Elevated MED1 levels promote EMT and cancer stem cell formation, thereby contributing to tumor aggressiveness and therapy resistance [54,55]. Functionally, MED1 serves as a signal-responsive nuclear receptor coactivator whose association with the Mediator complex is enhanced by MAPK–ERK-dependent phosphorylation, which promotes interaction with MED7 and potentiates hormone-dependent RNA polymerase II transcription [56].

#### 4.2.2. Mediator Complex Subunit 4 (MED4)

Relatively little is known about the role of MED4 in carcinogenesis. One study associated MED4 deletion and downregulation with poor clinical outcomes in cervical cancer (CC) and identified MED4 as a driver of chemo-radiotherapy resistance [57]. MED4 has also been identified as a survival gene in retinoblastoma (Rb) [58]. Functionally, MED4 forms a conserved heterodimer with MED9 that constitutes a central structural element of the Mediator middle module, contributing to a flexible scaffold that supports RNA polymerase II regulation and may influence cellular vulnerability to transcriptional stress [59].

#### 4.2.3. Mediator Complex Subunit 7 (MED7)

MED7 serves as a significant prognostic marker in BCa, particularly in estrogen receptor-positive (ER^+^) luminal subtypes, where higher MED7 expression correlates with improved survival [60]. In contrast, MED7 is upregulated in hepatocellular carcinoma (HCC) and contributes to tumor progression [61], highlighting context-dependent functions. Structurally, MED7 forms a highly extended heterodimer with MED21 in the Mediator middle module, creating a flexible hinge that connects the middle and head modules and enables large-scale conformational changes during transcriptional regulation [62]. Consistent with this architectural role, integrity of the MED7–MED21 hinge is required for stable Mediator–Pol II holoenzyme assembly, as hinge-disrupting mutations selectively impair Pol II binding without destabilizing core Mediator [63]. In addition, the N-terminal region of MED7 forms a distinct submodule with MED31 that positively regulates transcription of specific gene sets, demonstrating that MED7 contributes both to global Mediator architecture and to gene-selective transcriptional control [64].

#### 4.2.4. Mediator Complex Subunit 9 (MED9)

Recent studies have linked MED9 to human disease, with the short isoform of MED9 being strongly upregulated in familial dilated cardiomyopathy, suggesting a role in cardiac pathology [65]. Functionally, MED9 partners with MED4 to form a central heterodimer within the Mediator middle module that contributes to the extended and flexible core architecture of Mediator, positioning MED9 as a structural stabilizer that may influence Pol II regulation under conditions of altered transcriptional demand [59].

#### 4.2.5. Mediator Complex Subunit 10 (MED10)

MED10 has been implicated in multiple disease contexts, including bladder cancer [66] and HCC [67]. In addition, MED10 has been identified among Alzheimer’s disease (AD) risk-associated genes, pointing to broader roles beyond cancer [68]. Functionally, MED10 occupies a flanking position within the Mediator middle module adjacent to the MED7–MED21 heterodimer, where it contributes to middle-module flexibility and may facilitate dynamic communication between core Mediator architecture and RNA polymerase II during regulated transcription [59].

#### 4.2.6. Mediator Complex Subunit 19 (MED19)

MED19 is consistently upregulated across a wide spectrum of malignancies—including HCC [69], Prostate Ca [70], Breast Ca [71], Colorectal Ca [72], and Bladder Ca [73]—where it promotes tumor cell proliferation, migration, and resistance to therapy [69,70,71,72,73]. Functionally, MED19 directly binds Hox homeodomain transcription factors through a conserved animal-specific motif and acts as a Pol II-embedded cofactor required for Hox-dependent developmental gene activation, providing a direct molecular interface between sequence-specific transcription factors and the general transcription machinery [74]. Consistent with a broader architectural role, loss of MED19 (Rox3) in yeast destabilizes functional coupling of the Mediator Middle module with the Head and Tail, impairing Pol II binding, CTD phosphorylation by TFIIH, and activated transcription, thereby highlighting MED19 as a key conduit linking activators to the core transcriptional apparatus [75].

#### 4.2.7. Mediator Complex Subunit 21 (MED21)

MED21 is a highly conserved core component of the Mediator middle module that forms an extended heterodimer with MED7, creating a flexible hinge composed of a four-helix bundle and coiled-coil elements that spans a substantial portion of the Mediator complex and enables conformational rearrangements during transcriptional regulation [62]. Structural and biochemical analyses demonstrate that integrity of the MED7–MED21 hinge is essential for Mediator–RNA polymerase II holoenzyme assembly, as hinge-disrupting mutations selectively impair Pol II binding and increase middle-module disorder without destabilizing the core complex [63]. Consistent with this essential architectural role, no monogenic human disorder has yet been directly attributed to pathogenic MED21 variants, likely reflecting the strong evolutionary constraint imposed by its requirement for Mediator structural integrity and Pol II engagement.

#### 4.2.8. Mediator Complex Subunit 26 (MED26)

MED26 functions as a regulatory adaptor that coordinates the transition from transcription initiation to elongation within the Mediator complex. Structural and biochemical analyses show that MED26 promotes RNA polymerase II (Pol II) CTD engagement by antagonizing CDK8 kinase module-mediated inhibition, a process that can be triggered by nuclear receptor binding to reconfigure Mediator and enable MED26-dependent Pol II interaction [76]. Beyond initiation, MED26 selectively recruits elongation machineries, including the Super Elongation Complex (SEC) and Little Elongation Complex (LEC), thereby regulating gene-type-specific elongation and transcription termination of protein-coding, snRNA, and replication-dependent histone genes [18]. Consistent with this regulatory role, no monogenic human disorder has been directly attributed to pathogenic MED26 variants to date, although altered MED26 expression has been reported in several cancers.

#### 4.2.9. Mediator Complex Subunit 31 (MED31)

MED31 regulates self-renewal and adipogenic differentiation of human mesenchymal stem cells [77]. Notably, a study of Parkinson’s disease (PD) reported sex-biased expression of MED31, with significantly increased levels observed in the frontal cortex of female patients [78]. Functionally, MED31 forms a conserved middle-module submodule with the N-terminal region of MED7 that is required for activated RNA polymerase II transcription and selectively regulates defined gene sets, including those involved in metabolic pathways, providing a structural context for its cell type- and state-dependent functions [64].

Together, these findings underscore the dual nature of the Mediator middle module. While some subunits function primarily as disease-associated regulatory cofactors, others play indispensable roles in maintaining the transcriptional architecture required for Pol II function. This functional heterogeneity further reinforces the concept that Mediator dysregulation operates at the level of individual subunits and interfaces, rather than through wholesale disruption of the complex.

### 4.3. Tail Module

The Mediator tail module comprises mediator complex subunits MED14, MED15, MED16, MED23, MED24, and MED25. This module is positioned at the interface between transcription factors and the core Mediator architecture, enabling signal-responsive regulation of gene expression.

#### 4.3.1. Mediator Complex Subunit 14 (MED14)

Among Mediator subunits, MED14 is unique in its dual role as a central structural scaffold and a functional regulator of Pol II engagement. MED14 coordinates interactions between multiple Mediator subunits and enhances Mediator–Pol II association, thereby stabilizing the overall architecture of the complex [9]. A recent study further demonstrated that the N-terminal half of MED14 is essential for Pol II interaction and for efficient recruitment of Pol II to gene promoters [79]. Beyond its general role in transcription, specific *med14* mutant alleles have been linked to impaired neural crest development [80] and defects in stem cell maintenance in zebrafish [81].

#### 4.3.2. Mediator Complex Subunit 15 (MED15)

MED15 has emerging relevance in cardiovascular disease biology. A 2025 systems-level review identified MED15 as a regulatory node in lipid metabolism, inflammatory signaling, and oxidative stress, acting through SREBP, NF-κB, and NRF2 pathways that are central to the pathogenesis of atherosclerosis and coronary artery disease [82]. In cancer, MED15 contributes to tumor progression and metastatic dissemination in renal cell carcinoma (RCC) [83,84] and head and neck squamous cell carcinoma (HNSCC) [85]. Notably, phosphorylation of MED15 at threonine 603 (T603) functions as a molecular switch controlling senescence-associated secretory phenotype production, implicating MED15 as a novel regulator of tissue aging and cognitive decline [86]. Structurally, MED15 is a metazoan-enriched Tail-module subunit that engages extensively with the Mediator core, as revealed by mammalian cryo-EM analyses, positioning it to modulate Mediator conformation and activator-dependent transcriptional programs [47].

#### 4.3.3. Mediator Complex Subunit 16 (MED16)

Biallelic variants in MED16 have been identified in 25 individuals presenting with neurodevelopmental delay and ID. These findings position MED16 among a growing group of genes associated with so-called “MEDopathies,” a term describing neurodevelopmental disorders caused by mutations in Mediator subunit genes [87,88]. Structurally, MED16 is a metazoan-enriched Tail-module subunit that interacts extensively with the Mediator core, as revealed by mammalian cryo-EM analyses, positioning it to influence Mediator conformation and activator-dependent transcription [47].

#### 4.3.4. Mediator Complex Subunit 23 (MED23)

MED23 plays both mechanistic and pathophysiological roles in transcriptional regulation. Loss-of-function and missense variants in MED23 (including R617Q) are associated with ID, epilepsy, and widespread dysregulation of gene expression. Mechanistically, MED23 mutations reprogram enhancer activity and chromatin architecture, underscoring its importance in transcriptional network stability [89,90]. Beyond transcriptional control, MED23 directly interfaces with mRNA processing machinery through interaction with hnRNP L, enabling Mediator-dependent regulation of alternative splicing and alternative polyadenylation and providing a molecular link between transcription and mRNA processing [91].

#### 4.3.5. Mediator Complex Subunit 24 (MED24)

MED24 has been identified as a critical oncogenic target of erb-b2 receptor tyrosine kinase 2 (ERBB2) in lung tumorigenesis. Elevated MED24 expression correlates with poor survival in patients with non-small cell lung cancer (NSCLC), suggesting that MED24 represents a promising therapeutic target, particularly in ERBB2-mutant tumors [92]. Structurally, MED24 is a metazoan-specific Tail-module subunit that engages extensively with the Mediator core, as revealed by mammalian cryo-EM analyses, positioning it to modulate Mediator conformation and activator-dependent transcriptional responses [47].

#### 4.3.6. Mediator Complex Subunit 25 (MED25)

A homozygous missense mutation in MED25 (p.Tyr39Cys) has been identified as the cause of severe ID characterized by congenital eye defects, growth retardation, and additional developmental abnormalities, highlighting the essential role of MED25 in human development [93]. Functionally, MED25 acts as a transcriptional coactivator that directly engages the activation domain of ATF6α, serving as a docking site for Mediator recruitment to ER stress-responsive promoters and enabling Pol II-dependent transcription during the unfolded protein response [94].

Collectively, these findings illustrate that the Mediator tail module serves as a signal-integration interface that couples transcription factor inputs to core Mediator architecture. Disease-associated alterations in tail subunits frequently disrupt transcriptional responsiveness rather than basal Pol II function, leading to context-dependent pathologies ranging from neurodevelopmental disorders to cancer, cardiovascular disease, and aging-related dysfunction. The enrichment of regulatory phosphorylation sites, activator-binding surfaces, and disease-associated variants within tail subunits underscores their potential as selective therapeutic entry points for modulating transcriptional programs without globally impairing Mediator integrity.

### 4.4. Kinase Module

The CDK8/19 kinase module (CKM) is a reversibly associated subcomplex of Mediator that regulates gene expression primarily through phosphorylation of transcription factors (TFs) and Mediator subunits. Increasing evidence implicates CKM components in a wide range of diseases, particularly cancer. The kinase module comprises cyclin-dependent kinase 8 (CDK8) or its paralog cyclin-dependent kinase 19 (CDK19), mediator complex subunit 12 (MED12 and its paralog MED12L), mediator complex subunit 13 (MED13 and MED13L), and cyclin C (CCNC). Disease associations linked to individual subunits are summarized below.

#### 4.4.1. Cyclin-Dependent Kinase 8 and 19 (CDK8/CDK19)

CDK8 and its paralog CDK19 function as the catalytic kinases of the Mediator kinase module (CKM), forming an evolutionarily conserved complex with cyclin C that associates with the RNA polymerase II holoenzyme and relays growth- and signal-dependent cues to the transcriptional machinery [95].

CDK8 and CDK19 play central roles in signal-dependent transcriptional regulation. In inflammation-associated gastric cancer (GCa), the miR-26b-5p–PDE4B/CDK8–STAT3 feedback loop has been identified as a critical regulatory circuit. By directly targeting phosphodiesterase 4B (PDE4B) and CDK8, miR-26b-5p suppresses STAT3 phosphorylation and nuclear translocation, thereby restraining gastric cancer cell proliferation [96]. In breast cancer (BCa), CDK8 expression and that of its interacting genes show strong correlation, supporting their utility as prognostic biomarkers [97].

In prostate cancer (PCa), CDK8 and CDK19 contribute to disease progression. Pharmacological inhibition of CDK8/CDK19 enhances tumor cell sensitivity to androgen deprivation while reducing migratory potential, highlighting these kinases as therapeutic targets [98]. The selective CDK8/CDK19 inhibitor cortistatin A (CA) has provided key mechanistic insights, demonstrating that kinase inhibition activates super-enhancer-associated genes in acute myeloid leukemia (AML) and disrupts phosphorylation networks involving DNA-binding TFs, chromatin-associated proteins, DNA repair factors, and Pol II. These findings extend CKM function beyond transcription to DNA repair and cellular metabolism [99,100,101]. Consistent with this, inhibition of CDK8/CDK19 suppresses solid tumor growth, including PCa [99,100,101].

Mechanistically, phosphorylation of signal transducer and activator of transcription 1 (STAT1) at serine 727 by the Mediator kinase stimulates AML cell proliferation in conjunction with JAK–STAT signaling. In contrast, CA-mediated inhibition of this phosphorylation induces growth arrest, reduces colony formation in patient-derived samples, and lowers leukemic allele burden in mouse models, underscoring the therapeutic potential of CKM inhibition [102].

#### 4.4.2. Cyclin C (CCNC)

CCNC plays a direct role in cardiac stress responses. Under stress conditions, CCNC translocates from the nucleus to mitochondria, where it modulates mitochondrial dynamics and cardiac function [103]. In oncology, CCNC contributes to B-cell acute lymphoblastic leukemia (ALL) progression by suppressing p53-mediated stress responses, thereby promoting leukemogenesis [104]. At the molecular level, CCNC forms a highly specific complex with CDK8 within the Mediator kinase module, as revealed by crystal structures that define a unique cyclin-recognition interface and an unconventional mode of CDK8 activation independent of activation-loop phosphorylation, providing a structural basis for CKM-dependent transcriptional regulation and pharmacological targeting [105].

#### 4.4.3. Mediator Complex Subunit 12 (MED12)

Pathogenic mutations in MED12 underlie a spectrum of X-linked ID syndromes, including FG, Lujan–Fryns, and Ohdo syndromes, as well as non-syndromic ID in hemizygous males. In females, de novo MED12 missense variants have been linked to Hardikar syndrome without ID [106]. MED12 mutations have also been identified in multiple cancers, including chronic lymphocytic leukemia (CLL) [107], uterine leiomyomas, and PCa [108]. Importantly, MED12 mutation status has been proposed as a predictive biomarker for response to gonadotropin-releasing hormone agonists in uterine leiomyoma [109] and colorectal cancer (CRC) [110]. Over the past decade, MED12 dysregulation has emerged as a key driver of breast fibroepithelial tumors, with associated alterations in WNT, TGF-β, and thyroid hormone receptor alpha (THRA) signaling pathways [111]. Mechanistically, MED12 acts as a regulatory subunit of the Mediator kinase module by wrapping its N-terminal region around the CDK8–Cyclin C complex to position an activation helix that stimulates CDK8 kinase activity, while cancer-associated mutations in this region alter CDK8 activation and transcriptional outputs without disrupting complex assembly [112].

#### 4.4.4. Mediator Complex Subunit 13 and MED13L (MED13/MED13L)

MED13 was initially linked to severe congenital heart defects [113]. MED13L haploinsufficiency syndrome is now recognized as a distinct neurodevelopmental disorder characterized by global developmental delay, ID, hypotonia, speech impairment, and variable brain MRI abnormalities. Foundational studies by Asadollahi and Adegbola and colleagues established this syndrome through analyses of copy number variants affecting MED13L [114,115]. Subsequent work has expanded the role of the MED13/MED13L axis to cardiac and metabolic regulation, with variants also implicated in developmental and epileptic encephalopathies [116].

Mechanistically, MED13 serves as the principal tether linking the CDK8 kinase module to the Mediator Tail/leg domain, enabling MED12–MED13-dependent repression by blocking RNA polymerase II recruitment and transcriptional reinitiation independently of CDK8 kinase activity [11].

Collectively, these findings establish the kinase module as a signal-responsive regulatory arm of the Mediator complex that integrates extracellular cues with transcriptional, metabolic, and DNA repair programs. Disease-associated alterations in CKM components frequently operate through aberrant phosphorylation networks rather than disruption of core Mediator architecture. The demonstrated pharmacological tractability of CDK8/CDK19, combined with the pleiotropic disease relevance of MED12, MED13, and CCNC, positions the kinase module as one of the most promising Mediator-derived targets for therapeutic intervention. Importantly, selective modulation of kinase module activity offers a strategy to reshape pathological transcriptional states while preserving essential basal transcription.

**Table 1:** Disease- and Function-Associated Landscape of Human Mediator Complex Subunits. Abbreviations are the same as those used in the legend of Figure 2.

Mediator Module	Subunit	Core Molecular Function	Representative Disease Associations
Head module	MED6	Signal transduction, Regulation of RNA Pol II [29,30,31]	LUAD [28]
	MED8	PIC formation, Coupling factor [34,35]	HCC [32], RCC [33]
	MED11	PIC stabilization [37]	ND [36]
	MED17	Transcription activation [40]	ID [38,39]
	MED20	PIC formation [34]	ND [41]
	MED22	PIC stabilization [37]	RF [42], CBT [43], HCC [44]
	MED27	Interaction with core Mediator [47], Structural stability [48]	NDS [45,46]
	MED28	Interaction with core Mediator [47]	EEL [49]
	MED29	Interaction with core Mediator [47]	PCH [50], OSCC [51], PaCa [52]
	MED30	Interaction with core Mediator [47], Structural stability [53]	CHD [53]
Middle module	MED1	Association with the meditor [56]	BC [54,55]
	MED4	Structural integrity [59]	CC [57], Rb [58]
	MED7	Mediator integrity and activated Pol II transcription [62] Holoenzyme assembly [63], Mediator architecture [64]	BC [60], HCC [61]
	MED9	Structural integrity [59]	CP [65]
	MED10	Structural integrity [59]	BlCa [66], HCC [67], AD [68]
	MED19	PolII co-factor [74], Intermodule interactions [75]	HCC [69], PCa [70], BC [71], CRC [72], BlCa [73]
	MED21	Mediator integrity and activated Pol II transcription [62], Holoenzyme assembly [63]	No established monogenic human disorder
	MED26	Transcriptional elongation and termination [18], Transcriptional regulation [76]	No established monogenic human disorder
	MED31	Mediator architecture [64]	PD [78]
Tail module	MED14	Structural stability [9], Pol II interaction [79]	NCD [80]
	MED15	Tail–core interactions [47]	CVD [82], RCC [83,84], HNSCC [85]
	MED16	Lower tail subunit interaction [47]	NDD and ID [87,88]
	MED23	Transcriptional control [91]	ID, EPI [89,90]
	MED24	Structural conformation [47]	NSLC [92]
	MED25	Mediator recruitment to genes [94]	ID [93]
Kinase module	CDK8/19	Signal-responsive transcriptional kinase [95]	GC [96], BC [97], PCa [98], AML [99]
	Cyclin C	Activates CDK8/19 [105]	B-ALL [104]
	MED12/12L	Activates CDK8 [112]	XLID [106], CLL [107], PCa [108], CRC [110]
	MED13/13L	Module tethering [11]	CHD [113], NDS [114,115]

## 5. Therapeutic Implications and Future Directions

Over the past decade, the Mediator complex—long appreciated as a central coordinator of transcription—has emerged as a compelling therapeutic target. In particular, research has converged on specific Mediator modules and subunits whose dysregulation contributes to disease, revealing actionable vulnerabilities rather than global Mediator failure. Below, we discuss major therapeutic strategies that have gained momentum in recent years and consider future directions for translating Mediator biology into clinical intervention.

### 5.1. Targeting the Mediator Kinase Module

Among Mediator components, the kinase module has attracted the greatest attention as a druggable entry point, as summarized in Table 2. In prostate cancer (PCa), recent studies have identified MED12 as a potential therapeutic vulnerability, suggesting that selective modulation of this subunit may offer new options for a disease that remains challenging to treat [117]. Beyond tumor cells, the kinase module also plays a critical role in shaping immune responses. Deletion of MED12 or CCNC markedly enhances effector T-cell expansion, cytokine production, metabolic fitness, and persistence under chronic stimulation—properties that are highly desirable for antitumor immunity [118].

Encouragingly, advances in genome-editing technologies, including CRISPR–Cas9, zinc-finger nucleases, TALENs, and base editors, are rapidly moving toward clinical application, making therapeutic manipulation of kinase module components increasingly feasible [119]. A major inflection point for the field was the discovery that the natural product Cortistatin A (CA) is a highly selective inhibitor of CDK8 and CDK19. This landmark finding established that Mediator kinase activity is not only druggable but also biologically consequential in disease models [100]. Subsequent medicinal chemistry efforts have yielded potent, orally bioavailable CDK8 inhibitors with promising activity in AML [120].

The therapeutic window for targeting the Mediator kinase module is inherently nuanced. CDK8 and CDK19 are required for efficient induction of signal-responsive transcriptional programs, supporting their appeal as targets in signaling-driven cancers. However, sustained inhibition of CDK8/19 kinase activity elicits adaptive responses, including post-transcriptional upregulation of core Mediator and kinase-module components, potentially reshaping basal transcriptional states. Importantly, while small-molecule kinase inhibitors suppress catalytic activity, they preserve kinase-independent scaffolding functions such as Cyclin C stabilization, whereas PROTAC-based degraders eliminate both enzymatic and non-enzymatic functions, offering a mechanistically distinct and potentially more durable mode of pathway suppression. These considerations argue for carefully tuned dosing, temporal control, or combination strategies to limit compensatory transcriptional rewiring while maximizing therapeutic efficacy [121].

### 5.2. Systemic Toxicity, Off-Target Liabilities, and Context-Dependent Responses to CDK8/19 Inhibition

Several CDK8/19 inhibitors have been associated with distinct forms of systemic toxicity. Chen et al. compared multiple compounds, including Cmpd3 (CCT251921), Cmpd4 (MSC2530818), Senexin B, 16-didehydro-cortistatin A (dCA), and 15w, and found that only Cmpd4 caused severe developmental toxicity in zebrafish, despite similar nanomolar CDK8/19 inhibitory potency among compounds. Kinome profiling revealed that Cmpd3 and Cmpd4 exhibited off-target kinase activities, suggesting that unintended kinase inhibition contributed to toxicity [122]. In contrast, Clarke et al. reported that two highly selective CDK8/19 inhibitors (Cmpd3 and Cmpd4) caused dose-limiting systemic toxicity in mice, including effects on intestinal epithelium, immune compartments, and stem-cell-associated tissues, consistent with on-pathway disruption of essential transcriptional programs [123].

### 5.3. MED19 as a Context-Dependent Oncogenic Dependency Within the Mediator Complex

An emerging therapeutic strategy in transcription-targeted cancer therapy focuses on selectively perturbing disease-relevant functions of individual Mediator subunits rather than globally inhibiting the entire complex. Although Mediator acts as an integrated transcriptional coactivator, increasing evidence indicates that specific subunits can become preferentially engaged by oncogenic signaling pathways, thereby sustaining pathological transcriptional programs while remaining partially dispensable for basal gene expression. This functional asymmetry creates opportunities to target Mediator subunits that operate as context-dependent transcriptional dependencies in cancer.

Within this framework, MED19 has emerged as a particularly compelling candidate. Across multiple tumor types, MED19 plays a disproportionate role in sustaining proliferative and invasive phenotypes and represents a potential therapeutic target, as summarized in Table 2. In non-small cell lung cancer, MED19 depletion induces G0/G1 cell-cycle arrest and enhances sensitivity to cisplatin-induced apoptosis, implicating MED19 in proliferative control and chemoresistance [124]. MED19 (also known as LCMR1) has also been linked to melanoma invasion, where it regulates *TSPAN8* expression to promote loss of cell–matrix adhesion, increased invasion, and tumorigenicity; notably, MED19/LCMR1 and *TSPAN8* expression correlate in melanoma lesions and are jointly suppressed by BRAF inhibition, positioning MED19 downstream of RAF–MEK–ERK signaling [125].

Consistent oncogenic roles for MED19 have been reported in additional malignancies. In bladder cancer, MED19 is overexpressed and correlates with advanced stage and high histopathological grade, while its depletion suppresses tumor growth and migration by attenuating Wnt/β-catenin signaling [73]. Similarly, MED19 is upregulated in breast cancer cells, where RNAi-mediated silencing induces apoptosis and reduces cell migration and invasion [126]. Collectively, these findings identify MED19 as a recurrent oncogenic dependency across diverse cancer contexts.

Taken together, these observations position MED19 as a promising therapeutic node within the Mediator complex. Defining the molecular interactions and regulatory mechanisms that confer MED19-specific oncogenic functions will be critical for the rational development of MED19-targeted strategies in precision oncology.

**Table 2:** Therapeutic and Oncogenic Targeting of the Mediator Complex. This table summarizes pharmacological and genetic strategies targeting the Mediator kinase module and the Mediator subunit MED19 across multiple cancer types. It integrates evidence on expression patterns, experimental perturbations, affected cellular phenotypes, implicated signaling pathways, and key references, highlighting both therapeutic opportunities and context-dependent oncogenic vulnerabilities within the Mediator complex.

Mediator Component	Drug/Modality	Disease Context	Mechanism	Key Reference
CDK8/CDK19	Cortistatin A (CA), Synthetic CDK8 inhibitors	AML; signaling-driven cancers	suppression of signal-induced transcription	[101,120]
CDK8/CDK19	PROTAC	AML	Degaradation of CDK8/CDK19 via PROTAC	[121]
MED12	Genetic perturbation (siMED12)	Prostate cancer	Reduced expression of AR and c-MYC	[117]
MED12/CCNC	Genetic deletion (CRISPR)	GD2+ (disialoganglioside) tumor cells	Cancer immunotherapy models	[118]
MED19	Genetic knockdown/depletion	Non-small cell lung cancer (NSCLC)	G0/G1 arrest; increased cisplatin sensitivity	[124]
MED19	Genetic suppression (RNAi)	Melanoma	Reduced proliferation and migration	[125]
MED19	Genetic suppression (RNAi)	Bladder cancer	Impaired tumor growth and invasion	[73]
MED19	Genetic suppression (RNAi)	Breast cancer	Disrupted oncogenic signaling	[126]

## 6. Perspectives and Future Directions

A decade of intensive research has firmly established the Mediator complex as more than a passive transcriptional scaffold. Instead, it represents a dynamic regulatory hub whose modular organization creates selective vulnerabilities exploitable for therapy. Realizing this potential will require a multidisciplinary strategy that integrates structural and chemical biology, multi-omics-based patient profiling, and rational combination therapies designed to target tumor-specific dependencies while sparing essential transcriptional programs in normal cells.

Emerging technologies are accelerating this transition. Artificial intelligence-based protein interaction modeling, coupled with large-scale CRISPR–Cas screening, is enabling the identification of regulatory “hotspots” within Mediator–nuclear receptor pathways. These computational approaches can also be applied to clinical datasets to support patient stratification and therapeutic personalization. A deeper understanding of how Mediator interfaces with nuclear receptors and other signaling pathways may ultimately yield more precise treatments for cancer, metabolic disease, and neurological disorders [127].

At the same time, continued integration of biochemical and biophysical assays with live-cell imaging, genome-wide profiling, and computational modeling is steadily advancing our understanding of Mediator function. The high degree of evolutionary conservation across species enables complementary use of diverse model systems: yeast remains invaluable for dissecting PPIs [128] and conserved regulatory mechanisms [129], while whole-organism models such as knockout mice provide essential insights into tissue-specific and physiological contexts [130].

Looking forward, a central challenge will be to place Mediator within a broader regulatory framework that extends beyond transcriptional activation alone. Accumulating evidence points to important roles for Mediator in chromatin organization, genome stability, and the coordination of signaling networks, suggesting that its contributions to disease may be both more extensive and more context-dependent than previously appreciated. Defining how individual subunits and modules participate in these processes—often in a cell type- or stimulus-specific manner—will be critical for understanding disease mechanisms and identifying actionable therapeutic nodes.

Future progress will therefore depend not only on deeper mechanistic dissection of individual Mediator components, but also on integrative strategies that bridge molecular detail with physiological and clinical relevance. As experimental and computational tools continue to mature, Mediator research is well positioned to move from descriptive models toward predictive and translational frameworks, ultimately enabling the rational targeting of Mediator-dependent vulnerabilities in human disease.

## Data Availability

This study is a review article and does not report any new data. All data discussed in this manuscript are available in the cited primary sources and published literature.

## References

[1] Nonet M.L., Young R.A. (1989). Intragenic and extragenic suppressors of mutations in the heptapeptide repeat domain of Saccharomyces cerevisiae RNA polymerase II. Genetics.

[2] Kelleher R.J., Flanagan P.M., Kornberg R.D. (1990). A novel mediator between activator proteins and the RNA polymerase II transcription apparatus. Cell.

[3] Thompson C.M., Koleske A.J., Chao D.M., Young R.A. (1993). A multisubunit complex associated with the RNA polymerase II CTD and TATA-binding protein in yeast. Cell.

[4] Kim Y.J., Bjorklund S., Li Y., Sayre M.H., Kornberg R.D. (1994). A multiprotein mediator of transcriptional activation and its interaction with the C-terminal repeat domain of RNA polymerase II. Cell.

[5] Richter W.F., Nayak S., Iwasa J., Taatjes D.J. (2022). The Mediator complex as a master regulator of transcription by RNA polymerase II. Nat. Rev. Mol. Cell Biol..

[6] Tsai K.L., Tomomori-Sato C., Sato S., Conaway R.C., Conaway J.W., Asturias F.J. (2014). Subunit Architecture and Functional Modular Rearrangements of the Transcriptional Mediator Complex. Cell.

[7] Yin J.W., Wang G. (2014). The Mediator complex: A master coordinator of transcription and cell lineage development. Development.

[8] Nozawa K., Schneider T.R., Cramer P. (2017). Core Mediator structure at 3.4 A extends model of transcription initiation complex. Nature.

[9] Cevher M.A., Shi Y., Li D., Chait B.T., Malik S., Roeder R.G. (2014). Reconstitution of active human core Mediator complex reveals a critical role of the MED14 subunit. Nat. Struct. Mol. Biol..

[10] Plaschka C., Lariviere L., Wenzeck L., Seizl M., Hemann M., Tegunov D., Petrotchenko E.V., Borchers C.H., Baumeister W., Herzog F. (2015). Architecture of the RNA polymerase II-Mediator core initiation complex. Nature.

[11] Knuesel M.T., Meyer K.D., Bernecky C., Taatjes D.J. (2009). The human CDK8 subcomplex is a molecular switch that controls Mediator coactivator function. Genes. Dev..

[12] Zhang H., Chen D.H., Mattoo R.U.H., Bushnell D.A., Wang Y., Yuan C., Wang L., Wang C., Davis R.E., Nie Y. (2021). Mediator structure and conformation change. Mol. Cell.

[13] Chen X., Yin X., Li J., Wu Z., Qi Y., Wang X., Liu W., Xu Y. (2021). Structures of the human Mediator and Mediator-bound preinitiation complex. Science.

[14] Schilbach S., Hantsche M., Tegunov D., Dienemann C., Wigge C., Urlaub H., Cramer P. (2017). Structures of transcription pre-initiation complex with TFIIH and Mediator. Nature.

[15] El Khattabi L., Zhao H., Kalchschmidt J., Young N., Jung S., Van Blerkom P., Kieffer-Kwon P., Kieffer-Kwon K.R., Park S., Wang X. (2019). A Pliable Mediator Acts as a Functional Rather Than an Architectural Bridge between Promoters and Enhancers. Cell.

[16] Maalouf C.A., Alberti A., Soutourina J. (2024). Mediator complex in transcription regulation and DNA repair: Relevance for human diseases. DNA Repair..

[17] Takahashi H., Parmely T.J., Sato S., Tomomori-Sato C., Banks C.A., Kong S.E., Szutorisz H., Swanson S.K., Martin-Brown S., Washburn M.P. (2011). Human mediator subunit MED26 functions as a docking site for transcription elongation factors. Cell.

[18] Takahashi H., Ranjan A., Chen S., Suzuki H., Shibata M., Hirose T., Hirose H., Sasaki K., Abe R., Chen K. (2020). The role of Mediator and Little Elongation Complex in transcription termination. Nat. Commun..

[19] Sabari B.R., Dall’Agnese A., Boija A., Klein I.A., Coffey E.L., Shrinivas K., Abraham B.J., Hannett N.M., Zamudio A.V., Manteiga J.C. (2018). Coactivator condensation at super-enhancers links phase separation and gene control. Science.

[20] Cho W.K., Spille J.H., Hecht M., Lee C., Li C., Grube V., Cisse I.I. (2018). Mediator and RNA polymerase II clusters associate in transcription-dependent condensates. Science.

[21] Boija A., Klein I.A., Sabari B.R., Dall’Agnese A., Coffey E.L., Zamudio A.V., Li C.H., Shrinivas K., Manteiga J.C., Hannett N.M. (2018). Transcription Factors Activate Genes through the Phase-Separation Capacity of Their Activation Domains. Cell.

[22] Luyties O., Taatjes D.J. (2022). The Mediator kinase module: An interface between cell signaling and transcription. Trends Biochem. Sci..

[23] Soutourina J. (2018). Transcription regulation by the Mediator complex. Nat. Rev. Mol. Cell Biol..

[24] Kagey M.H., Newman J.J., Bilodeau S., Zhan Y., Orlando D.A., van Berkum N.L., Ebmeier C.C., Goossens J., Rahl P.B., Levine S.S. (2010). Mediator and cohesin connect gene expression and chromatin architecture. Nature.

[25] Ramasamy S., Aljahani A., Karpinska M.A., Cao T.B.N., Velychko T., Cruz J.N., Lidschreiber M., Oudelaar A.M. (2023). The Mediator complex regulates enhancer-promoter interactions. Nat. Struct. Mol. Biol..

[26] Pugacheva E.M., Kubo N., Loukinov D., Tajmul M., Kang S., Kovalchuk A.L., Strunnikov A.V., Zentner G.E., Ren B., Lobanenkov V.V. (2020). CTCF mediates chromatin looping via N-terminal domain-dependent cohesin retention. Proc. Natl. Acad. Sci. USA.

[27] Whyte W.A., Orlando D.A., Hnisz D., Abraham B.J., Lin C.Y., Kagey M.H., Rahl P.B., Lee T.I., Young R.A. (2013). Master transcription factors and mediator establish super-enhancers at key cell identity genes. Cell.

[28] Yang C., Cheng D., Wang S., Wang B., Li Y., Wang G., Wang X., Shi C., Tian Y., Zhu K. (2025). Identification of the role of MED6 in the development and prognosis of lung adenocarcinoma based on multi-omics profiling. J. Cancer.

[29] Robinson P.J., Bushnell D.A., Trnka M.J., Burlingame A.L., Kornberg R.D. (2012). Structure of the mediator head module bound to the carboxy-terminal domain of RNA polymerase II. Proc. Natl. Acad. Sci. USA.

[30] Lee Y.C., Min S., Gim B.S., Kim Y.J. (1997). A transcriptional mediator protein that is required for activation of many RNA polymerase II promoters and is conserved from yeast to humans. Mol. Cell Biol..

[31] Lee Y.C., Kim Y.J. (1998). Requirement for a functional interaction between mediator components Med6 and Srb4 in RNA polymerase II transcription. Mol. Cell Biol..

[32] Jin X., Song Y., An Z., Wu S., Cai D., Fu Y., Zhang C., Chen L., Tang W., Zheng Z. (2022). A Predictive Model for Prognosis and Therapeutic Response in Hepatocellular Carcinoma Based on a Panel of Three MED8-Related Immunomodulators. Front. Oncol..

[33] Syring I., Klümper N., Offermann A., Braun M., Deng M., Boehm D., Queisser A., von Mässenhausen A., Brägelmann J., Vogel W. (2016). Comprehensive analysis of the transcriptional profile of the Mediator complex across human cancer types. Oncotarget.

[34] Larivière L., Geiger S., Hoeppner S., Röther S., Strässer K., Cramer P. (2006). Structure and TBP binding of the Mediator head subcomplex Med8-Med18-Med20. Nat. Struct. Mol. Biol..

[35] Chaves R.S., Herrero P., Moreno F. (1999). Med8, a subunit of the mediator CTD complex of RNA polymerase II, directly binds to regulatory elements of *SUC2* and *HXK2* genes. Biochem. Biophys. Res. Commun..

[36] Cali E., Lin S.J., Rocca C., Sahin Y., Al Shamsi A., El Chehadeh S., Chaabouni M., Mankad K., Galanaki E., Efthymiou S. (2022). A homozygous MED11 C-terminal variant causes a lethal neurodegenerative disease. Genet. Med..

[37] Seizl M., Larivière L., Pfaffeneder T., Wenzeck L., Cramer P. (2011). Mediator head subcomplex Med11/22 contains a common helix bundle building block with a specific function in transcription initiation complex stabilization. Nucleic Acids Res..

[38] Terabayashi T., Hashimoto S. (2021). Increased unfolded protein responses caused by MED17 mutations. Neurogenetics.

[39] Kaufmann R., Straussberg R., Mandel H., Fattal-Valevski A., Ben-Zeev B., Naamati A., Shaag A., Zenvirt S., Konen O., Mimouni-Bloch A. (2010). Infantile cerebral and cerebellar atrophy is associated with a mutation in the MED17 subunit of the transcription preinitiation mediator complex. Am. J. Hum. Genet..

[40] Kikuchi Y., Umemura H., Nishitani S., Iida S., Fukasawa R., Hayashi H., Hirose Y., Tanaka A., Sugasawa K., Ohkuma Y. (2015). Human mediator MED17 subunit plays essential roles in gene regulation by associating with the transcription and DNA repair machineries. Genes Cells.

[41] Vodopiutz J., Schmook M.T., Konstantopoulou V., Plecko B., Greber-Platzer S., Creus M., Seidl R., Janecke A.R. (2015). MED20 mutation associated with infantile basal ganglia degeneration and brain atrophy. Eur. J. Pediatr..

[42] Rodriguez P.Q., Unnersjo-Jess D., Zambrano S.S., Guo J., Moller-Hackbarth K., Blom H., Jahnukainen T., Ebarasi L., Patrakka J. (2020). Inactivation of mediator complex protein 22 in podocytes results in intracellular vacuole formation, podocyte loss and premature death. Sci. Rep..

[43] Lv Y., Gu G., Zeng R., Liu Z., Wu J., Zheng Y. (2023). Proteomics analysis of carotid body tumor revealed potential mechanisms and molecular differences among Shamblin classifications. Exp. Biol. Med..

[44] Wang W., Zhang C., Yu Q., Zheng X., Yin C., Yan X., Liu G., Song Z. (2021). Development of a novel lipid metabolism-based risk score model in hepatocellular carcinoma patients. BMC Gastroenterol..

[45] Maroofian R., Kaiyrzhanov R., Cali E., Zamani M., Zaki M.S., Ferla M., Tortora D., Sadeghian S., Saadi S.M., Abdullah U. (2023). Biallelic MED27 variants lead to variable ponto-cerebello-lental degeneration with movement disorders. Brain.

[46] Li X., Yiliyaer N., Guo T., Zhao H., Lei Y., Gu S. (2025). The indispensable role of Mediator complex subunit 27 during neurodevelopment. Cell Biosci..

[47] Zhao H., Young N., Kalchschmidt J., Lieberman J., El Khattabi L., Casellas R., Asturias F.J. (2021). Structure of mammalian Mediator complex reveals Tail module architecture and interaction with a conserved core. Nat. Commun..

[48] Zhu S., Chen Z., Liu C., Duong J., Tran T., Liang Z., Fang X., Ouyang K. (2024). The essential role of MED27 in stabilizing the mediator complex for cardiac development and function. Life Sci..

[49] Li L., Walsh R.M., Wagh V., James M.F., Beauchamp R.L., Chang Y.S., Gusella J.F., Hochedlinger K., Ramesh V. (2015). Mediator Subunit Med28 Is Essential for Mouse Peri-Implantation Development and Pluripotency. PLoS ONE.

[50] Arkush L., van Woerden G.M., Ziv L., Marek-Yagel D., Fonseca R., Breve E., Barel O., Shalva N., Veber A., Anikster Y. (2025). Biallelic MED29 variants cause pontocerebellar hypoplasia with cataracts. Eur. J. Hum. Genet..

[51] Huang S., Zhang J., Qiao Y., Pathak J.L., Zou R., Piao Z., Xie S., Liang J., Ouyang K. (2024). CHRDL1 inhibits OSCC metastasis via MAPK signaling-mediated inhibition of MED29. Mol. Med..

[52] Kuuselo R., Savinainen K., Sandstrom S., Autio R., Kallioniemi A. (2011). MED29, a component of the mediator complex, possesses both oncogenic and tumor suppressive characteristics in pancreatic cancer. Int. J. Cancer.

[53] Tan C., Zhu S., Chen Z., Liu C., Li Y.E., Zhu M., Zhang Z., Zhang Z., Zhang L., Gu Y. (2021). Mediator complex proximal Tail subunit MED30 is critical for Mediator core stability and cardiomyocyte transcriptional network. PLoS Genet..

[54] Leonard M., Zhang X. (2019). Estrogen receptor coactivator Mediator Subunit 1 (MED1) as a tissue-specific therapeutic target in breast cancer. J. Zhejiang Univ. Sci. B.

[55] Yang Y., Leonard M., Luo Z., Yeo S., Bick G., Hao M., Cai C., Charif M., Wang J., Guan J.L. (2021). Functional cooperation between co-amplified genes promotes aggressive phenotypes of HER2-positive breast cancer. Cell Rep..

[56] Belakavadi M., Pandey P.K., Vijayvargia R., Fondell J.D. (2008). MED1 phosphorylation promotes its association with mediator: Implications for nuclear receptor signaling. Mol. Cell Biol..

[57] Lando M., Holden M., Bergersen L.C., Svendsrud D.H., Stokke T., Sundfor K., Glad I.K., Kristensen G.B., Lyng H. (2009). Gene dosage, expression, and ontology analysis identifies driver genes in the carcinogenesis and chemoradioresistance of cervical cancer. PLoS Genet..

[58] Dehainault C., Garancher A., Castera L., Cassoux N., Aerts I., Doz F., Desjardins L., Lumbroso L., Montes de Oca R., Almouzni G. (2014). The survival gene MED4 explains low penetrance retinoblastoma in patients with large RB1 deletion. Hum. Mol. Genet..

[59] Larivière L., Plaschka C., Seizl M., Petrotchenko E.V., Wenzeck L., Borchers C.H., Cramer P. (2013). Model of the Mediator middle module based on protein cross-linking. Nucleic Acids Res..

[60] Joseph C., Macnamara O., Craze M., Russell R., Provenzano E., Nolan C.C., Diez-Rodriguez M., Sonbul S.N., Aleskandarany M.A., Green A.R. (2018). Mediator complex (MED) 7: A biomarker associated with good prognosis in invasive breast cancer, especially ER+ luminal subtypes. Br. J. Cancer.

[61] Chen Z.L., Ma Y.Y., Mou X.Z., Zhang J.G. (2023). Upregulation of MED7 was associated with progression in hepatocellular carcinoma. Cancer Biomark..

[62] Baumli S., Hoeppner S., Cramer P. (2005). A conserved mediator hinge revealed in the structure of the MED7·MED21 (Med7·Srb7) heterodimer. J. Biol. Chem..

[63] Sato S., Tomomori-Sato C., Tsai K.L., Yu X., Sardiu M., Saraf A., Washburn M.P., Florens L., Asturias F.J., Conaway R.C. (2016). Role for the MED21-MED7 Hinge in Assembly of the Mediator-RNA Polymerase II Holoenzyme. J. Biol. Chem..

[64] Koschubs T., Seizl M., Larivière L., Kurth F., Baumli S., Martin D.E., Cramer P. (2009). Identification, structure, and functional requirement of the Mediator submodule Med7N/31. EMBO J..

[65] Franzese M., Zanfardino M., Soricelli A., Coppola A., Maiello C., Salvatore M., Schiano C., Napoli C. (2024). Familial Dilated Cardiomyopathy: A Novel MED9 Short Isoform Identification. Int. J. Mol. Sci..

[66] Wu C.C., Wang Y.H., Hu S.W., Wu W.L., Yeh C.T., Bamodu O.A. (2021). MED10 Drives the Oncogenicity and Refractory Phenotype of Bladder Urothelial Carcinoma Through the Upregulation of hsa-miR-590. Front. Oncol..

[67] Liu J., Lv Y., Liu K., Li Z., Chen B., Bu Y. (2025). MED10 as a Novel Oncogenic Driver in HCC: Promoting Cell Cycle Progression and Proliferation Through RAF1 Activation. Front. Biosci. (Landmark Ed.).

[68] Gao L., Li J., Yan M., Aili M. (2021). Methylation factor MRPL15 identified as a potential biological target in Alzheimer’s disease. Aging.

[69] Zhang Y., Qin P., Xu X., Li M., Huang H., Yan J., Zhou Y. (2021). Mediator Complex Subunit 19 Promotes the Development of Hepatocellular Carcinoma by Regulating the AKT/mTOR Signaling Pathway. Front. Oncol..

[70] Imberg-Kazdan K., Ha S., Greenfield A., Poultney C.S., Bonneau R., Logan S.K., Garabedian M.J. (2013). A genome-wide RNA interference screen identifies new regulators of androgen receptor function in prostate cancer cells. Genome Res..

[71] Zhang X., Gao D., Fang K., Guo Z., Li L. (2019). Med19 is targeted by miR-101-3p/miR-422a and promotes breast cancer progression by regulating the EGFR/MEK/ERK signaling pathway. Cancer Lett..

[72] Ye H., Li W., Wu K., Liu Y., Lv Y., Zhu Y., Luo H., Cui L. (2020). The SP1-Induced Long Noncoding RNA, LINC00339, Promotes Tumorigenesis in Colorectal Cancer via the miR-378a-3p/MED19 Axis. Onco Targets Ther..

[73] Yuan H., Yu S., Cui Y., Men C., Yang D., Gao Z., Zhu Z., Wu J. (2017). Knockdown of mediator subunit Med19 suppresses bladder cancer cell proliferation and migration by downregulating Wnt/β-catenin signalling pathway. J. Cell Mol. Med..

[74] Boube M., Hudry B., Immarigeon C., Carrier Y., Bernat-Fabre S., Merabet S., Graba Y., Bourbon H.M., Cribbs D.L. (2014). Drosophila melanogaster Hox transcription factors access the RNA polymerase II machinery through direct homeodomain binding to a conserved motif of mediator subunit Med19. PLoS Genet..

[75] Baidoobonso S.M., Guidi B.W., Myers L.C. (2007). Med19(Rox3) regulates Intermodule interactions in the Saccharomyces cerevisiae mediator complex. J. Biol. Chem..

[76] Zhao H., Li J., Xiang Y., Malik S., Vartak S.V., Veronezi G.M.B., Young N., Riney M., Kalchschmidt J., Conte A. (2024). An IDR-dependent mechanism for nuclear receptor control of Mediator interaction with RNA polymerase II. Mol. Cell.

[77] Beadle E.P., Straub J.A., Bunnell B.A., Newman J.J. (2018). MED31 involved in regulating self-renewal and adipogenesis of human mesenchymal stem cells. Mol. Biol. Rep..

[78] Lopez-Cerdan A., Andreu Z., Hidalgo M.R., Grillo-Risco R., Catala-Senent J.F., Soler-Saez I., Neva-Alejo A., Gordillo F., de la Iglesia-Vaya M., Garcia-Garcia F. (2022). Unveiling sex-based differences in Parkinson’s disease: A comprehensive meta-analysis of transcriptomic studies. Biol. Sex. Differ..

[79] Baris Y., Jabbar J., Yozgat Y., Dinccelik-Aslan M., Cigirgan E., Erden M., Bay S., Aslan V., Cevher M.A. (2025). N-terminal half of MED14 is critical for Mediator-RNA polymerase II interaction and the resulting transcription. J. Biol. Chem..

[80] Lou X., Burrows J.T., Scott I.C. (2015). Med14 cooperates with brg1 in the differentiation of skeletogenic neural crest. BMC Dev. Biol..

[81] Burrows J.T., Pearson B.J., Scott I.C. (2015). An in vivo requirement for the mediator subunit med14 in the maintenance of stem cell populations. Stem Cell Rep..

[82] Schiano C., Napoli C. (2025). Mediator complex: Update of key insights into transcriptional regulation of ancestral framework and its role in cardiovascular diseases. Eur. J. Med. Res..

[83] Hua X., Ge S., Zhang L., Jiang Q., Chen J., Xiao H., Liang C. (2024). MED15 is upregulated by HIF-2alpha and promotes proliferation and metastasis in clear cell renal cell carcinoma via activation of SREBP-dependent fatty acid synthesis. Cell Death Discov..

[84] Weiten R., Muller T., Schmidt D., Steiner S., Kristiansen G., Muller S.C., Ellinger J., Syring I. (2018). The Mediator complex subunit MED15, a promoter of tumour progression and metastatic spread in renal cell carcinoma. Cancer Biomark..

[85] Adler D., Offermann A., Halbach R., Vogel W., Braun M., Kristiansen G., Bootz F., Wenzel J., Mikut R., Lengerke C. (2015). Clinical and molecular implications of MED15 in head and neck squamous cell carcinoma. Am. J. Pathol..

[86] Li H., Zheng Y., Yuan C., Wang J., Zhao X., Yang M., Xiong D., Yang Y., Dai Y., Gao Y. (2025). A phosphorylation switch in the Mediator MED15 controls cellular senescence and cognitive decline. Cell Discov..

[87] Guillouet C., Agostini V., Baujat G., Cocciadiferro D., Pippucci T., Lesieur-Sebellin M., Georget M., Schatz U., Fauth C., Louie R.J. (2025). Bi-allelic MED16 variants cause a MEDopathy with intellectual disability, motor delay, and craniofacial, cardiac, and limb malformations. Am. J. Hum. Genet..

[88] Huang Y., Xiang Z., Xiang Y., Pan H., He M., Guo Z., Kanca O., Liu C., Zhang Z., Zhan H. (2025). Biallelic MED16 variants disrupt neural development and lead to an intellectual disability syndrome. J. Genet. Genom..

[89] Trehan A., Brady J.M., Maduro V., Bone W.P., Huang Y., Golas G.A., Kane M.S., Lee P.R., Thurm A., Gropman A.L. (2015). MED23-associated intellectual disability in a non-consanguineous family. Am. J. Med. Genet. A.

[90] Yang Y., Li C., Chen Z., Zhang Y., Tian Q., Sun M., Zhang S., Yu M., Wang G. (2023). An intellectual disability-related MED23 mutation dysregulates gene expression by altering chromatin conformation and enhancer activities. Nucleic Acids Res..

[91] Huang Y., Li W., Yao X., Lin Q.J., Yin J.W., Liang Y., Heiner M., Tian B., Hui J., Wang G. (2012). Mediator complex regulates alternative mRNA processing via the MED23 subunit. Mol. Cell.

[92] Liu J., Wang T., Willson C.J., Janardhan K.S., Wu S.P., Li J.L., DeMayo F.J. (2019). ERBB2 Regulates MED24 during Cancer Progression in Mice with Pten and Smad4 Deletion in the Pulmonary Epithelium. Cells.

[93] Basel-Vanagaite L., Smirin-Yosef P., Essakow J.L., Tzur S., Lagovsky I., Maya I., Pasmanik-Chor M., Yeheskel A., Konen O., Orenstein N. (2015). Homozygous MED25 mutation implicated in eye-intellectual disability syndrome. Hum. Genet..

[94] Sela D., Conkright J.J., Chen L., Gilmore J., Washburn M.P., Florens L., Conaway R.C., Conaway J.W. (2013). Role for human mediator subunit MED25 in recruitment of mediator to promoters by endoplasmic reticulum stress-responsive transcription factor ATF6α. J. Biol. Chem..

[95] Tassan J.P., Jaquenoud M., Léopold P., Schultz S.J., Nigg E.A. (1995). Identification of human cyclin-dependent kinase 8, a putative protein kinase partner for cyclin C. Proc. Natl. Acad. Sci. USA.

[96] Xu T., Xie M., Jing X., Jiang H., Wu X., Wang X., Shu Y. (2023). Loss of miR-26b-5p promotes gastric cancer progression via miR-26b-5p-PDE4B/CDK8-STAT3 feedback loop. J. Transl. Med..

[97] Broude E.V., Gyorffy B., Chumanevich A.A., Chen M., McDermott M.S., Shtutman M., Catroppo J.F., Roninson I.B. (2015). Expression of CDK8 and CDK8-interacting Genes as Potential Biomarkers in Breast Cancer. Curr. Cancer Drug Targets.

[98] Offermann A., Joerg V., Becker F., Roesch M.C., Kang D., Lemster A.L., Tharun L., Behrends J., Merseburger A.S., Culig Z. (2022). Inhibition of Cyclin-Dependent Kinase 8/Cyclin-Dependent Kinase 19 Suppresses Its Pro-Oncogenic Effects in Prostate Cancer. Am. J. Pathol..

[99] Pelish H.E., Liau B.B., Nitulescu I.I., Tangpeerachaikul A., Poss Z.C., Da Silva D.H., Caruso B.T., Arefolov A., Fadeyi O., Christie A.L. (2015). Mediator kinase inhibition further activates super-enhancer-associated genes in AML. Nature.

[100] Nakamura A., Nakata D., Kakoi Y., Kunitomo M., Murai S., Ebara S., Hata A., Hara T. (2018). CDK8/19 inhibition induces premature G1/S transition and ATR-dependent cell death in prostate cancer cells. Oncotarget.

[101] Poss Z.C., Ebmeier C.C., Odell A.T., Tangpeerachaikul A., Lee T., Pelish H.E., Shair M.D., Dowell R.D., Old W.M., Taatjes D.J. (2016). Identification of Mediator Kinase Substrates in Human Cells using Cortistatin A and Quantitative Phosphoproteomics. Cell Rep..

[102] Nitulescu I.I., Meyer S.C., Wen Q.J., Crispino J.D., Lemieux M.E., Levine R.L., Pelish H.E., Shair M.D. (2017). Mediator Kinase Phosphorylation of STAT1 S727 Promotes Growth of Neoplasms With JAK-STAT Activation. EBioMedicine.

[103] Ponce J.M., Coen G., Spitler K.M., Dragisic N., Martins I., Hinton A., Mungai M., Tadinada S.M., Zhang H., Oudit G.Y. (2020). Stress-Induced Cyclin C Translocation Regulates Cardiac Mitochondrial Dynamics. J. Am. Heart Assoc..

[104] Trifinopoulos J., List J., Klampfl T., Klein K., Prchal-Murphy M., Witalisz-Siepracka A., Bellutti F., Fava L.L., Heller G., Stummer S. (2025). Cyclin C promotes development and progression of B-cell acute lymphoblastic leukemia by counteracting p53-mediated stress responses. Haematologica.

[105] Schneider E.V., Böttcher J., Blaesse M., Neumann L., Huber R., Maskos K. (2011). The structure of CDK8/CycC implicates specificity in the CDK/cyclin family and reveals interaction with a deep pocket binder. J. Mol. Biol..

[106] Plassche S.V., Brouwer A.P. (2021). MED12-Related (Neuro)Developmental Disorders: A Question of Causality. Genes.

[107] Kampjarvi K., Jarvinen T.M., Heikkinen T., Ruppert A.S., Senter L., Hoag K.W., Dufva O., Kontro M., Rassenti L., Hertlein E. (2015). Somatic MED12 mutations are associated with poor prognosis markers in chronic lymphocytic leukemia. Oncotarget.

[108] Kampjarvi K., Kim N.H., Keskitalo S., Clark A.D., von Nandelstadh P., Turunen M., Heikkinen T., Park M.J., Makinen N., Kivinummi K. (2016). Somatic MED12 mutations in prostate cancer and uterine leiomyomas promote tumorigenesis through distinct mechanisms. Prostate.

[109] Nagai K., Asano R., Sekiguchi F., Asai-Sato M., Miyagi Y., Miyagi E. (2023). MED12 mutations in uterine leiomyomas: Prediction of volume reduction by gonadotropin-releasing hormone agonists. Am. J. Obs. Gynecol..

[110] Siraj A.K., Masoodi T., Bu R., Pratheeshkumar P., Al-Sanea N., Ashari L.H., Abduljabbar A., Alhomoud S., Al-Dayel F., Alkuraya F.S. (2018). MED12 is recurrently mutated in Middle Eastern colorectal cancer. Gut.

[111] Lae M., Gardrat S., Rondeau S., Richardot C., Caly M., Chemlali W., Vacher S., Couturier J., Mariani O., Terrier P. (2016). MED12 mutations in breast phyllodes tumors: Evidence of temporal tumoral heterogeneity and identification of associated critical signaling pathways. Oncotarget.

[112] Klatt F., Leitner A., Kim I.V., Ho-Xuan H., Schneider E.V., Langhammer F., Weinmann R., Müller M.R., Huber R., Meister G. (2020). A precisely positioned MED12 activation helix stimulates CDK8 kinase activity. Proc. Natl. Acad. Sci. USA.

[113] Muncke N., Jung C., Rüdiger H., Ulmer H., Roeth R., Hubert A., Goldmuntz E., Driscoll D., Goodship J., Schön K. (2003). Missense mutations and gene interruption in PROSIT240, a novel TRAP240-like gene, in patients with congenital heart defect (transposition of the great arteries). Circulation.

[114] Adegbola A., Musante L., Callewaert B., Maciel P., Hu H., Isidor B., Picker-Minh S., Le Caignec C., Delle Chiaie B., Vanakker O. (2015). Redefining the MED13L syndrome. Eur. J. Hum. Genet..

[115] Asadollahi R., Oneda B., Sheth F., Azzarello-Burri S., Baldinger R., Joset P., Latal B., Knirsch W., Desai S., Baumer A. (2013). Dosage changes of MED13L further delineate its role in congenital heart defects and intellectual disability. Eur. J. Hum. Genet..

[116] Trivisano M., De Dominicis A., Micalizzi A., Ferretti A., Dentici M.L., Terracciano A., Calabrese C., Vigevano F., Novelli G., Novelli A. (2022). MED13 mutation: A novel cause of developmental and epileptic encephalopathy with infantile spasms. Seizure.

[117] Guzman J., Hart M., Weigelt K., Neumann A., Aigner A., Andolfi C., Handle F., Rheinheimer S., Fischer U., Immel U.D. (2025). The MicroRNA miR-454 and the mediator complex component MED12 are regulators of the androgen receptor pathway in prostate cancer. Sci. Rep..

[118] Freitas K.A., Belk J.A., Sotillo E., Quinn P.J., Ramello M.C., Malipatlolla M., Daniel B., Sandor K., Klysz D., Bjelajac J. (2022). Enhanced T cell effector activity by targeting the Mediator kinase module. Science.

[119] Ashmore-Harris C., Fruhwirth G.O. (2020). The clinical potential of gene editing as a tool to engineer cell-based therapeutics. Clin. Transl. Med..

[120] Chen Z., Wang Q., Yan Y.Y., Jin D., Wang Y., Zhang X.X., Liu X.H. (2024). Discovery of novel and potent CDK8 inhibitors for the treatment of acute myeloid leukaemia. J. Enzym. Inhib. Med. Chem..

[121] Chen M., Li J., Zhang L., Wang L., Cheng C., Ji H., Altilia S., Ding X., Cai G., Altomare D. (2023). CDK8 and CDK19: Positive regulators of signal-induced transcription and negative regulators of Mediator complex proteins. Nucleic Acids Res..

[122] Chen M., Li J., Liang J., Thompson Z.S., Kathrein K., Broude E.V., Roninson I.B. (2019). Systemic Toxicity Reported for CDK8/19 Inhibitors CCT251921 and MSC2530818 Is Not Due to Target Inhibition. Cells.

[123] Clarke P.A., Ortiz-Ruiz M.J., TePoele R., Adeniji-Popoola O., Box G., Court W., Czasch S., El Bawab S., Esdar C., Ewan K. (2016). Assessing the mechanism and therapeutic potential of modulators of the human Mediator complex-associated protein kinases. eLife.

[124] Wei L., Wang X.W., Sun J.J., Lv L.Y., Xie L., Song X.R. (2015). Knockdown of Med19 suppresses proliferation and enhances chemo-sensitivity to cisplatin in non-small cell lung cancer cells. Asian Pac. J. Cancer Prev. APJCP.

[125] Agaesse G., Barbollat-Boutrand L., Sulpice E., Bhajun R., El Kharbili M., Berthier-Vergnes O., Degoul F., de la Fouchardiere A., Berger E., Voeltzel T. (2017). A large-scale RNAi screen identifies LCMR1 as a critical regulator of Tspan8-mediated melanoma invasion. Oncogene.

[126] Dong Y., Liu Y., Zhao L., Qu C. (2021). Mediator complex subunit 19 regulates the proliferation, migration and invasion of human breast cancer cells. Trop. J. Pharm. Res..

[127] Zhou L., Zhao M., Zhai Y., Lin Q. (2025). The Mediator Complex: A Regulatory Hub for Transcriptional Activity of Nuclear Receptors. Cells.

[128] Laval F., Coppin G., Twizere J.C., Vidal M. (2023). Homo cerevisiae-Leveraging Yeast for Investigating Protein-Protein Interactions and Their Role in Human Disease. Int. J. Mol. Sci..

[129] Gastelum S., Michael A.F., Bolger T.A. (2023). Saccharomyces cerevisiae as a research tool for RNA-mediated human disease. Wiley Interdiscip. Rev. RNA.

[130] Ilchuk L.A., Kubekina M.V., Okulova Y.D., Silaeva Y.Y., Tatarskiy V.V., Filatov M.A., Bruter A.V. (2023). Genetically Engineered Mice Unveil In Vivo Roles of the Mediator Complex. Int. J. Mol. Sci..

